# Universal HCV Screening in Hospitalised Patients in France: It Could Be a Good Option! The DEVICHO Study

**DOI:** 10.1111/jvh.70038

**Published:** 2025-05-21

**Authors:** Si Ahmed Si Nafa, Souad Benali, Guillaume Penaranda, Sylvie Deuffic‐Burban, Magali Madau, Laurence Lecomte, Gaelle Valle, Sandrine Thibault, Constance Chailloux, Valérie Oules, Clara Dassetto, Floriane Sellier, Olivia Pietri, Paul Castellani, Xavier Adhoute, Marc Bourlière

**Affiliations:** ^1^ Department of Hepato‐Gastroenterology Hospital Saint Joseph Marseille France; ^2^ Laboratoire Alphabio – Biogroup Marseille France; ^3^ Université Paris Cité and Université Sorbonne Paris Nord, Inserm, IAME Paris France; ^4^ Department of Bacteriology and Virology Hospital Saint Joseph Marseille France; ^5^ INSERM UMR 1252 IRD SESSTIM, Aix Marseille Université Marseille France

**Keywords:** chronic hepatitis, HCV, HCV RNA, HCV‐Ab, hepatitis C, hospitalised patients, universal screening

## Abstract

In France, chronic hepatitis C whatever fibrosis stage or comorbidities can be freely treated by any physician. However, screening is still currently based on risk factors, and universal screening remains controversial. The aims of this prospective DEVICHO study were to assess the value of universal screening in hospitalised patients, to evaluate the prevalence of HCV infection and to compare the short‐term cost and benefit of this strategy with routine screening. From November 2019 to November 2021, all hospitalised patients from 22 departments were asked by their physicians to be tested for HCV. 4986/25,663 (19.4%) in the DEVICHO study (Group 1) and 1803 patients (7%) outside the study (Group 2) were screened. HCV screening rate varied widely (0%–75.1%) between departments. One hundred and ninety‐nine patients (2.9%) were HCV‐Ab positive. 29/199 HCV‐Ab positive patients (14.6%) or 29/6789 patients tested (0.4%) were HCV‐RNA positive. Among the 29 viremic patients, 9 (31%) were treated, all achieving sustained virological response, but two patients died rapidly after treatment. Seventeen patients died untreated within a year of diagnosis, and three patients were not treated. Universal screening compared to routine practice would be more expensive and more effective, resulting in an additional cost of €11,060 per HCV RNA infection identified and €36,600 per HCV cure, both below the GDP per capita of France (€38,000, Eurostat 2023). Even if the population screened is older, often with significant comorbidities, hospital‐based HCV screening is efficient because its prevalence is higher in hospitalised patients than in the general population. Additionally, this screening strategy appears to be cost effective. However, healthcare professionals and insufficient linkage to care are the main barriers to screening.

**Trial Registration:** ClinicalTrials.gov identifier: NTC 04437277

Chronic hepatitis C (CHC) remains a public health problem, with more than 50 million people infected worldwide [1]. CHC is currently a curable disease, with nearly 97% cured after a first‐line pangenotypic treatment and 96% cured with a second‐line rescue treatment [2, 3]. The World Health Organization (WHO) set the goal of hepatitis C elimination for 2030 [4]. However, there are a number of obstacles to achieving this objective, including the asymptomatic nature of the infection and the absence or failure of policies to encourage screening [5].

In 2014, the French general report on viral hepatitis proposed that screening for HCV, HBV and HIV should be quasi‐systematically combined, that the screening of at‐risk populations be strengthened, and lastly that routine testing for each of these three viruses should be offered at least once to men of 18–60 years old who have never been tested. Besides, HCV screening should be routinely performed during the first trimester of pregnancy [6].

Since 2017 in France, all HCV‐infected patients regardless of their fibrosis stage or comorbidities can all be treated. Since 2018, all physicians can prescribe HCV treatment and direct acting antivirals (DAAs) are available in all pharmacies [7]. Despite this open access to treatment, the number of treated patients has decreased since 2017 [8, 9]. This can be explained by at least two things: all patients diagnosed and followed up in the healthcare system have been treated and screening of at‐risk populations is far from optimal. In 2019 the French ‘Haute Autorité de Santé’ (HAS) recommend screening people according to a long list of risk factors [10]. However, a study using a Markov model demonstrated that in France universal screening was the most effective strategy for HCV and is cost effective when treatment is initiated regardless of fibrosis stage [11]. Nevertheless, studies of universal screening in countries with a low HCV prevalence show that such screening is not efficient and cost effective [12, 13, 14].

From 2014 to 2021, 25 million individuals (38.6% of the population) in France have been screened for HCV with regional disparities and 96,776 DAAs treatment have been initiated [8]. This number of diagnosed and treated patients represents only 66% of CHC in the French general population [15], which is lower than the WHO's 2020 target of 75% [4]. A study using multi‐parameter evidence synthesis suggests that the prevalence of HCV viraemic patients may be around 0.29% (0.16–0.46) in France in 2019, with around 1/3 attributed to PWID [5]. In 2023, more than 80,000 HCV‐infected patients remain untreated and partly undiagnosed. Therefore, HCV screening and linkage to care remain the main barriers to HCV elimination and become a challenging task that requires specific time‐consuming interventions.

To assess the value of universal HCV screening in all patients hospitalised in a tertiary general hospital, we decided to conduct a prospective study. Saint Joseph Hospital is a large hospital with more than 700 beds covering all medical and surgical fields except brain surgery. It includes a large maternity department with more than 5000 deliveries per year. In 2017, HCV screening was performed in 5532/80,784 (6%) hospitalised patients, and HCV antibodies (HCV‐Ab) prevalence was 1.6%, twice as high as in the general population.

The main objective of this prospective study was to update the prevalence of HCV infection in hospitalised patients and to study the implementation of this universal screening, its limitations, the management of HCV patients who were diagnosed and their linkage to care. Lastly, we aimed to compare the short‐term cost and benefit of this strategy compared to current practices.

## 1 Patients and Methods

Before starting the prospective study, a survey was carried out among all hospital doctors, except paediatricians, emergency physicians and biologists, between June and September 2019 to assess their knowledge of how hepatitis C is transmitted and how the disease is managed. Physicians were asked four questions: (1) do you screen your patients for HCV: always, never or if they have risk factors? (2) do you consider the following to be risk factors: former or current PWID, blood transfusion before 1990, patients with at‐risk sexual behaviour or a sexually transmitted disease (STD), patients over 50 years old, patients with at‐risk medical procedures before 1990, others? (3) in case of HCV‐Ab positivity do you ask for HCV RNA testing: yes or no? (4) do you refer HCV patients to a liver specialist: yes or no? Sixty‐eight per cent (181/267) of physicians from 27 different departments answered the questionnaire.

The prospective study began in November 2019 in 22 departments excluding emergency, paediatric, maternity and dialysis departments. All residents and physicians in the 22 departments were trained between September and November 2019 to offer hepatitis C screening to all patients entering the hospital. Once patients had signed the informed consent form, they were screened for HCV‐Ab and, if positive, HCV RNA was determined by reflex PCR. In the event of positive HCV RNA, patients were managed by the hepatology team (2 doctors and 2 coordinating nurses) and treated if necessary, depending on the other pathologies for which the patient was hospitalised. For each patient tested, the following data were collected: age, sex, weight, height, BMI and for HCV‐Ab positive patients, HCV risk factors, alcohol consumption and IV drug use. In HCV RNA‐positive patients, the fibrosis stage was assessed by either Fibro scan, Fibro test or FIB‐4 before treatment.

In addition, an information campaign was carried out in the hospital, with the distribution of flyers, posters in waiting rooms and a campaign on the hospital's television networks to explain the risks of CHC, the ways in which the virus is transmitted, the risks of contamination, as well as the excellent results and minimal side effects of DAA treatments.

Furthermore, during the course of the study, in agreement with the local ethics committee, we retrospectively collected data throughout the study period on the patients who had been screened for HCV and on the outcomes of the patients with positive HCV‐RNA who were screened outside the DEVICHO protocol.

The protocol was approved by the national ethics committee (2018‐A02595‐50, NTC 04437277) and the fees for all biological and virological tests were funded by the ‘Agence Régionale de Santé de Provence Alpes Cotes d'Azur’ (ARS PACA).

### 1.1 End Points

The principal end point was to prospectively assess the prevalence of HCV‐Ab and HCV RNA in the hospitalised population.

Secondary end points were: to analyse the baseline characteristics of the patients who entered the trial, to identify barriers to HCV screening, to evaluate the risk factors in all patients with positive HCV‐Ab, to analyse the linkage to care as well as HCV treatment and sustained response among positive HCV RNA patients. Moreover, we aimed to evaluate the mean cost of universal screening and its benefits compared to current practice in hospitalised patients.

### 1.2 Statistics

The demographic, morphometric and clinical variables of each patient have been described using descriptive statistics. These descriptive statistics have been calculated and expressed as median and IQR for quantitative data and as frequency and percentage for qualitative data. Seroprevalence of HCV‐Ab and viremia (HCV RNA) has been calculated and described using percentage and 95% confidence interval (95% CI). Comparisons between study groups have been performed using chi‐squared test or Fisher exact for qualitative data, and Student's *T*‐test or Wilcoxon test for quantitative data according to their type. Statistical significance was set to *α* < 0.05, unless otherwise indicated. Statistical analyses have been performed using SAS version 9.4 (SAS Institute Inc., Cary, NC, USA).

#### 1.2.1 Sample Size

An estimate made by the ‘Institut National de Veille Sanitaire’ (INVS) in 2011 in metropolitan France in subjects aged from 18 to 80 years found a seroprevalence of HCV Ab of 0.75% (95% CI: 0.62–0.92) of which 0.42% (95% CI: 0.33–0.53) with positive viremia [16]. A study using multi‐parameter evidence synthesis suggests that the prevalence of HCV viraemic patients may be around 0.29% (0.16–0.46) in France in 2019 [5].

The Saint Joseph Hospital's 2016 Activity Report mentions 65,000 admissions, which represent approximately 40,000 patients/year. We hope to obtain informed consent from at least 5000 patients. Under these conditions, considering that the hospital seroprevalence will likely be higher than that of the general population, testing 5000 patients would allow us to estimate a seroprevalence of 0.75% (95% CI: 0.49–1.01).

#### 1.2.2 Economic Analysis

**FIGURE 1:**
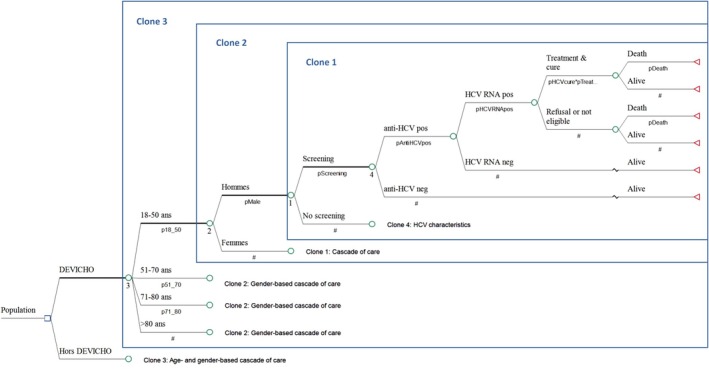
Decision tree of the population trajectory according to the two strategies. A clone indicates an exact duplicate of a tree structure to attach at other locations in the tree. A square represents a decision node, and branches emanating from a decision node represent the options. A circle represents a probability node, and the branches emanating from a probability node represent the different possible events whose probability of occurrence is indicated below (the hash mark corresponds to 1 minus the sum of the other probabilities emanating from the same node). A triangle is used to indicate a terminal node, that is, a final outcome. The probability values are estimated from the survey.

A short‐term economic analysis of universal screening (DEVICHO program) and current practice (outside DEVICHO) is conducted. A decision tree is built to evaluate the two screening strategies in the total cohort of 25,663 hospitalised patients over a year (Figure 1). Each strategy is evaluated in terms of effectiveness (expected numbers of HCV RNA infection identified and HCV cure) and cost (total cost among hospitalised patients). Two economic ratios are calculated: cost per additional HCV RNA infection identified and cost per additional HCV cure. A health service perspective is taken, with costs reported in 2024 euros (€).

The data collected during the trial are used to feed the decision tree: proportion of men and women according to four age groups (18–50, 51–70, 71–80 and > 80 years); completion of HCV screening, proportion of patients with positive HCV Ab among those screened and proportion of HCV RNA‐positive patients among those screened HCV Ab positive, according to the four age groups, sex and programme (DEVICHO or outside DEVICHO); linkage to care of HCV RNA‐positive patients (treatment, refusal/ineligibility) and the outcome over a year (survival with or without HCV cure, or death).

All direct costs from the time of HCV testing to DAA therapy initiation are calculated: screening tests, that is, HCV Ab (€11.61) and quantitative detection of HCV RNA (€54) for HCV‐Ab positive patients; tests for all HCV RNA‐positive patients, that is, HIV serodiagnosis (€11.34), Ag HBs serodiagnosis (€13.50), transaminase dosage (€2.43) and blood count (€5.40); hepatology consultation (€28), Fibro scan (€31.29) and abdominal ultrasound (€52.45) for all HCV RNA‐positive patients. Costs of first‐line treatment (Maviret or Epclusa, €24,000) and second‐line treatment in case of failure (Vosevi, €36,000) are also considered.

Sensitivity analysis was conducted varying parameter value either by ±20%, or between 0% and 5% for zero values.

## 2 Results

### 2.1 Pre‐Study Survey

The results of the survey carried out among doctors between June and September 2019 show that 23% never screen their patients for HCV, 14% always screen for HCV and 63% screen according to risk factors. If we remove the 17 midwives who always screen pregnant women, we have only 5% (8/164) of non‐liver specialists who always screen for HCV and 25% who never screen for HCV. Considering risk factors, 81% of these doctors' screen PWID, 73% in case of unsafe sexual practice or STD, 65% in case of blood transfusions before 1990, 39% in case of history of unsafe medical procedure before 1990 and 23% in patients over 50 years old. In case of HCV–Ab positivity, only 56% of non‐liver specialists ask for HCV RNA testing. Our survey demonstrates that among non‐liver specialists practicing in a tertiary hospital, 25% never screen for HCV and in those who screen according to risk factors, awareness about HCV transmission needs to be improved. An information campaign about hepatitis C was conducted in the 22 departments involved in the DEVICHO study between September and November 2019.

### 2.2 Flow Diagram of the Study

**FIGURE 2:**
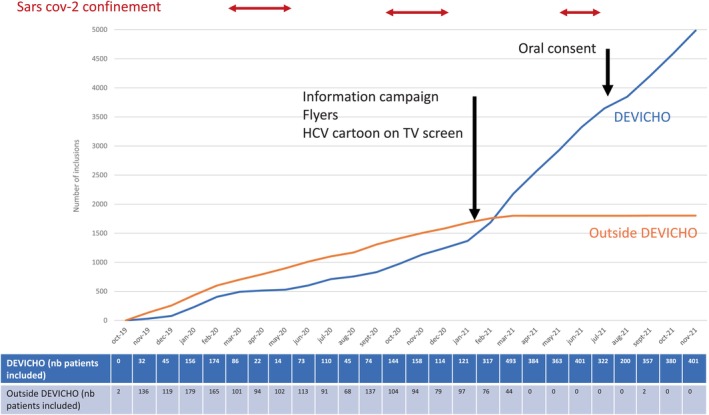
Flow diagram recruitment of the study according to SarS‐Cov2 pandemic confinement and ethics committee amendment.

The prospective DEVICHO study began in November 2019 with an initial goal of recruiting 5000 patients in 1 year, but the COVID‐19 epidemic had a strong impact on HCV screening. The three periods of confinement in 2020 and 2021 slowed recruitment and physician motivation waned. To face this problem, we re‐encouraged all involved doctors, ran a new information campaign and created a 5‐min cartoon dedicated to HCV, which was broadcasted on all the hospital's TV screens. In July 2021, the ethics committee authorised oral consent, which significantly increased recruitment (Figure 2).

Between November 2019 and November 2021, 25,663 patients were hospitalised in the 22 departments. 4986 patients (19.4%) were screened in the DEVICHO study (Group 1). Only one patient refused screening in the DEVICHO study.

During the same period, 1803 patients (7.0%) (Group 2) were screened outside the study, either systematically as part of screening protocols for certain surgical procedures or immunotherapy and/or chemotherapy treatments or on the basis of the existence of risk factors.

### 2.3 Baseline Characteristics

Overall, HCV screening was carried out in 6789 patients (26.4%). Table 1 shows the demographic characteristics of the patients. Patients screened inside and outside DEVICHO were of the same age as the general hospitalised population, but were more frequently male 55% versus 49% (*p* < 0.001). The percentage of HCV screening rate varied widely (0%–75.1%) between departments both within and outside the DEVICHO study (Table 2), reflecting the variable motivation of prescribers and the existence of screening protocols for certain surgical procedures or pre‐treatment check‐ups with immunotherapy or chemotherapy. Throughout the study period, caregiver motivation improved discreetly in the post‐COVID period, with almost exclusive screening in the DEVICHO study during the last 8 months.

**TABLE 1:** Characteristics of the population.

Characteristics	Hospitalised population without HCV screening (*N* = 18,874)	DEVICHO population (*N* = 4986)	*p*	Outside DEVICHO		*p*
				Systematic^a^ (*N* = 1423), 79%	With RF (*N* = 380), 21%	
Mean age (SD)	66.8 (18.6)	66.6 (15.5)	0.54	66.7 (16.7)	65.3 (15.8)	0.14
Sex, *N* (%)						
Men	9281 (49%)	2720 (55%)	< 0.001	754 (53%)	212 (56%)	0.33
Women	9593 (51%)	2266 (45%)		669 (47%)	168 (44%)	
HCV Ab, *N* (%)						
Positive	—	137 (2.7%)	—	27 (1.9%)	35 (9.2%)	< 0.001
Mean age (SD)		68.1 (12.9%)		72.9 (12.2%)	62.7 (11.7%)	0.002
Sex—men (%)		89 (65%)		14 (52%%)	23 (66%)	0.27
Negative		4849 (97.2%)		1396 (98.1%)	345 (90.8%)	—
Mean age (SD)		66.5 (15.6%)		66.6 (16.8%)	65.6 (16.2%)	0.31
Sex—men (%)		2631 (54%)		740 (53%)	189 (55%)	0.55
HCV RNA, *N* (%)						
Detectable	—	21 (15.3%)	—	2 (8%)	6 (18.2%)	0.45
Mean age (SD)		73 (13.4%)		85.5 (10.6%)	62.8 (15.3%)	NA
Sex—men (%)		15 (71.4%)		1 (50%)	5 (83%)	NA
Undetectable		116 (84.7%)		23 (92%)	27 (81.8%)	—
Mean age (SD)		67.2 (12.7%)		72.5 (11.8%)	61.8 (10.9%)	0.002
Sex—men (%)		74 (63.8%)		11 (47.8%)	17 (63%)	0.28
Not done		0		2	2	

Abbreviation: RF, risk factors.

^a^ Systematic screening is screening done systematically before some surgical procedure, before immunosuppressive therapies either in rheumatology, dermatology, oncology, etc.

**TABLE 2:** Screening rate, HCV Ab‐positive prevalence and HCV RNA‐positive prevalence according to department.

Department	*n*	Screening rate DEVICHO	Screening rate outside DEVICHO	Overall screening rate	HCV Ab+ (%)	HCV RNA+ (%)
Vascular surgery	1350	810 (60%)	25 (1.9%)	835 (61.9%)	29 (3.5)	6 (20.7)
Plastic surgery	57	0	0	0	0	0
Cardiac surgery	2317	550 (23.7)	249 (10.5%)	799 (34.5%)	13 (1.6)	0
Digestive surgery	1717	51 (3%)	23 (1.3%)	74 (4.3%)	1 (1.4)	0
Dermatology	266	136 (51.1%)	38 (14.3%)	174 (65.4%)	9 (5.2)	0
Endocrinology	931	306 (32.9%)	23 (2.5%)	329 (35.3%)	2 (0.6)	0
Cardiology	4689	384 (8.2%)	456 (9.7%)	840 (17.9%)	20 (2.4)	2 (10)
Geriatric	9	0	0	0	0	0
Gynaecology	736	3 (0.4%)	5 (0.7%)	8 (1.2%)	0	0
Hepato‐gastroenterology	1587	664 (41.8%)	155 (9.8%)	819 (51.6%)	53 (6.5)	9 (17)
Internal medicine	590	23 (3.9%)	0	23 (3.9%)	0	0
Post emergency medicine	1512	243 (16.1%)	176 (11.6%)	419 (27.7%)	10 (2.4)	1 (10)
Nephrology	247	0	0	0	0	0
Neurology	954	201 (21.1%)	91 (9.5%)	292 (30.6%)	10 (3.4)	2 (20)
Oncology	213	4 (1.9%)	156 (73.2%)	160 (75.1%)	9 (5.6)	2 (22.2)
Ophthalmology	146	2 (1.4%)	1 (0.7%)	3 (2.1%)	0	0
ENT	473	0	2 (0.4%)	2 (0.4%)	0	0
Orthopaedic surgery	1799	36 (2%)	14 (0.8%)	50 (2.8%)	1 (2)	0
Pneumology	2840	598 (21.1%)	80 (2.8%)	678 (23.9%)	15 (2.2)	4 (26.7)
Rheumatology	1418	792 (55.9%)	262 (18.5%)	1054 (74.4%)	22 (2.1)	2 (9.1)
Palliative care	335	0	5 (1.5%)	5 (1.5%)	0	0
Urology	1477	183 (12.4%)	42 (2.8%)	225 (15.2%)	5 (2.2)	1 (20)
	25,663	4986 (19.4%)	1803 (7%)	6789 (26.4%)	199 (2.9)	29 (0.4)

### 2.4 HCV‐Ab Prevalence

HCV‐Ab positivity was detected in 199 patients (2.9%): 137 (2.7%) among DEVICHO patients (Group 1) and 62 (3.4%) among non‐DEVCHO patients (Group 2). In the latter group, the prevalence of positive HCV‐Ab was significantly higher in patients screened for risk factors 35/380 (9.2%) than in those screened systematically 27/1423 (1.9%) *p* < 0.001. Patients HCV‐Ab positive, screened for the existence of a risk factor, were significantly younger: 62.7 versus 72.9 years (*p* = 0.002) in non‐DEVICHO patients, and 62.7 versus 68.1 years (*p* = 0.027) in DEVICHO patients. There was no difference in sex ratio. As expected, there is no information on alcohol consumption in the population outside DEVICHO. Overall, the prevalence of positive HCV‐Ab is more than three times higher than that observed in the general population.

Among DEVICHO patients (Table 3), the mean age was comparable between HCV‐Ab positive and negative patients (68.1 vs. 66.5 years, *p* = 0.81), but HCV‐Ab positive patients were more often male (65% vs. 54.3% *p* = 0.0131) and had a significantly lower BMI (24.7 vs. 26, *p* = 0.04). In HCV‐Ab positive patients, a risk factor for transmission was found in 78.1% (107/137) of patients. The high rate of blood transfusion and unsafe medical practice as risk factors can be explained by the advanced age of our HCV‐Ab positive population. Forty‐two patients (39.3%) had at‐risk alcohol consumption (20 g/day in women and 30 g/day in men).

**TABLE 3:** Demographic characteristics of the DEVICHO population according to HCV‐Ab status.

Parameter	DEVICHO screening	HCV‐Ab negative	HCV‐Ab positive	*p*
*N*	4986	4849 (97.2%)	137 (2.7%)	—
Mean age (SD)	66.6 (15.5)	66.5 (15.6)	68.1 (12.9)	0.8115
Sex, *N* (%)				
Men	2720 (55%)	2631 (54.3%)	89 (65.0%)	0.0131
Women	2266 (45%)	2218 (45.7%)	48 (35.0%)	
BMI	26.0 (9.3)	26.0 (9.4)	24.7 (5.7)	0.0434
Risk factors				
No			30 (21.9%)	
Yes			107 (78.1%)	
IVDU			39 (36.4%)	
Blood transfusion			30 (28.0%)	
Tattoo/piercing			17 (15.9%)	
Unsafe medical care			61 (57.0%)	
Sexual at‐risk factors			20 (18.7%)	
Stays in endemic countries			25 (23.4%)	
Prison stays			2 (1.9%)	
Psychiatric hospitalisation			3 (2.8%)	
At‐risk alcohol consumption			42 (39.3%)	

### 2.5 HCV RNA Prevalence

A positive HCV RNA was observed in 29/199 HCV‐Ab positive patients (14.6%) or 29/6789 patients tested (0.4%); 21 patients (15.3%) in the DEVICHO group and 8 patients (13.8%) in the non‐DEVICHO group. Because reflex testing was not done systematically on HCV‐Ab positive tests outside DEVICHO, 4/62 patients (6%) were not screened for HCV RNA in the non‐DEVICHO group.

Overall, the prevalence of positive HCV RNA (0.4%) is more than 1.4–4 times higher than what is observed in the general population.

Among DEVICHO patients (Table 4), the mean age was comparable between HCV RNA‐positive and negative patients (73 vs. 67.2, *p* = 0.10). Interestingly, 47.6% of HCV RNA‐positive patients were over 75 years, and 10% were over 90 years. There was no difference between HCV RNA‐positive and negative patients in terms of sex ratio, BMI or risk factors. Interestingly, the absence of risk factors was more frequent in viremic patients. As expected, advanced fibrosis was more frequent in viremic patients.

**TABLE 4:** Demographic characteristics of the DEVICHO population according to HCV RNA status.

Parameter	HCV RNA negative	HCV RNA positive	*p*
*N* = 137	116 (84.7%)	21 (15.3%)	—
Mean age (SD)	67.2 (12.7)	73.0 (13.4)	0.1007
Sex, *N* (%)			
Men	74 (63.8%)	15 (71.4%)	0.6222
Women	42 (36.2%)	6 (28.6%)	
BMI	25.0 (5.9)	23.3 (3.9)	0.0792
Risk factors			
No	23 (19.8%)	7 (33.3%)	
Yes	93 (80.2%)	14 (66.7%)	0.2486
IVDU	34 (36.6%)	5 (35.7%)	1.0000
Blood transfusion	26 (28.0%)	4 (28.6%)	1.0000
Tattoo/piercing	16 (17.2%)	1 (7.1%)	0.6791
Unsafe medical care	54 (58.1%)	7 (50.0%)	0.7501
Sexual at‐risk factors	20 (21.5%)	1 (7.1%)	0.6740
Stays in endemic countries	23 (24.7%)	2 (14.3%)	0.7190
Prison stays	2 (2.2%)	0	1.0000
Psychiatric hospitalisation	2 (2.2%)	1 (7.1%)	0.3067
At‐risk alcohol consumption	34 (36.6%)	8 (57.1%)	0.3959
HCV cured			
Spontaneously	25 (21.5%)		
After treatment	79 (68.1%)		
Unknown	12		
FIB‐4 status			
Means (SD)	3.54 (7.54)	6.19 (8.95)	0.0255
*N* (%)			
< 1.30	37 (32.7%)	3 (14.3%)	0.1419
1.30–2.67	39 (34.5%)	7 (33.3%)	
≥ 2.67	37 (32.7%)	11 (52.4%)	
Not available	3 (2.6%)		

Eighty‐five per cent of HCV‐Ab‐positive patients were cured, most often after being treated, reflecting the effectiveness of previous management since 2015, with access to treatment for all patients regardless of fibrosis stage since 2016, and available to all doctors starting in 2019.

### 2.6 Outcomes of Patients With Positive HCV RNA

The outcomes of HCV RNA‐positive patients are described in Table 5.

**TABLE 5:** Outcomes of HCV RNA‐positive patients in DEVICHO and outside DEVICHO.

Parameter	Fibrosis stage	Age (years)	Sex	Associated conditions at screening	HCV treated	Response	Status by end of 2023
DEVICHO patients							
pts# 1^a^	F4	60	M	Diffused arteritis	Yes	SVR	Alive
pts# 2^a^	F3/F4	59	M	Kidney angioplasty	Yes	SVR	Alive
pts# 3^a^	F2	95	F	Diffused arteritis	No		Death
pts# 4	F3	88	F	Diffuse arteritis and dementia	No		Death
pts# 5	F2	75	M	Poly metastatic lung cancer	No		Death
pts# 6^a^	F4	78	M	HCC (BCLC‐C) with portal thrombus	No		Death
pts# 7^a^	F4	56	M	HCC (BCLC‐B) ⇒ liver transplant	No		Death post LT
pts# 8	F0/F1	86	M	Extensive bladder cancer and Alzheimer's disease	No		Death
pts# 9^a^	F0/F1	70	M	Extensive uncontrolled bladder cancer	No		Death
pts# 10^a^	F3	68	M	HCC (BCLC‐B)	Yes	SVR	Alive
pts# 11^a^	F3	86	M	Lung and colon cancer	No		Death
pts# 12^a^	F4	52	M	Heart failure	Yes	SVR	Alive
pts# 13	F0	68	M	HCC (BCLC‐C)	No		Death
pts# 14	F4	88	F	Breast cancer with bone and liver metastasis	No		Death
pts# 15	F0/F1	80	M	Low back pain	Yes	SVR	Alive
pts# 16^a^	F4	60	F	HCC (BLCL‐D)	No		Death
pts# 17^a^	F0	68	M	LT 2007, severe CKD waiting for KT	Yes	SVR	Alive
pts# 18^a^	F3	81	M	HCC (BCLC‐D)	No		Death
pts# 19	F4	71	F	Metastatic pancreas cancer	No		Death
pts# 20^a^	F4	52	M	Variceal bleeding on cirrhosis	Refused		Alive
pts# 21	F2	92	F	Dementia	No		Death
Patients outside DEVICHO study							
pts# 1	F2	93	F	Hearth failure NYHAIII, no liver fibrosis	No		Alive
pts# 2	F2	78	M	Extended pancreatic cancer	No		Death
pts# 3	F4	59	M	Cirrhosis (CP‐A) + obstructive lung disease	Yes	SVR	Death
pts# 4	F4	50	M	HCC (BCLC‐D)	No		Death
pts# 5	F4	61	M	HCC (BCLC‐D)	No		Death
pts# 6	F0	55	M	Burger's disease	Yes	SVR	Alive
pts# 7	F0	59	M	Vascularitis, lung cancer	Yes	SVR	Death
pts# 8	F0	93	F	None	Refused		Alive

^a^ Aware of their HCV status before the study but not treated.

In the DEVICHO group, 6/21 patients were treated, and all achieved SVR. One patient with hepatocellular carcinoma (HCC) was transplanted and was to be treated, but died of infection early post‐transplant. Four patients with advanced HCC could not be treated and died rapidly of their liver disease. Nine patients were not treated because of their other diseases and all died. Finally, one cirrhotic patient refused treatment and is still alive. Interestingly, 13/21 (62%) of viremic patients knew their HCV status prior to the study, including 5/6 of treated patients.

In the non‐DEVICHO group, 3/8 patients were treated and achieved SVR 12, but two of these patients died within the year due to progression of lung cancer (pts#7) and multiple organ failure with cirrhosis (pts#3). Three patients were not treated because of their comorbidities (advanced HCC for two and pancreatic cancer for one patient). Finally, two patients over 90 years of age were not treated due to the absence of fibrosis and are still alive.

Overall, among the 29 viremic patients, 9 (31%) were treated, all achieving SVR12, but two patients died rapidly after treatment, one from extra‐hepatic cancer and the other from liver failure. Seventeen patients died untreated within a year of diagnosis, including seven with advanced HCC. Three patients were not treated, two because of refusal and one because of advanced age without hepatic fibrosis, and these patients are still alive. These results reflect the fact that patients diagnosed with HCV were often elderly and had associated pathologies that were rapidly lethal, whether hepatic (HCC) or extra‐hepatic.

### 2.7 Economic Analysis

In the total cohort of 25,663 patients hospitalised over 2 years, universal screening identified 21 HCV RNA infections, cured six of them and cost €231,400 (€9.02 per hospitalised patient screened). This overall cost can be broken down into four components: €57,900 for the HCV Ab testing, €7400 for HCV RNA quantification, €3000 for other blood tests and care for HCV RNA‐positive patients before treatment and €163,100 for HCV treatment.

By comparison, current practices identified eight HCV RNA infections, cured three of them and cost €87,500 (€3.41 per hospitalised patient screened): €20,900 for the HCV Ab testing, €3300 for HCV RNA quantification, €1200 for other blood tests and care for HCV RNA‐positive patients before treatment and €62,100 for HCV treatment.

Universal screening would be more expensive and more effective, resulting in an additional cost of €11,060 per HCV RNA infection identified and €36,600 per HCV cure, both below the GDP per capita of France (€38,000, Eurostat 2023). Our conclusion remained unchanged during sensitivity analysis.

## 3 Discussion

The main finding of our study is that hospital‐based screening is highly effective because the prevalence of HCV‐Ab positive patients in this hospitalised population is 2.7%, which is more than three times higher than that observed in the general population [12]. Similarly, the prevalence of viremic patients (0.4%) is more than 2–4 times higher than that observed in the general population [5, 12]. This shows that hospital‐based screening is a good opportunity for HCV screening. Interestingly, 21.9% of HCV‐Ab positive patients had no identified risk factor for HCV, which is an additional argument for systematic screening. Our results also suggest that hospital‐based universal screening, even if more expensive (€9.02 vs. €3.41 per hospitalised patient screened), is also more effective and may be cost effective compared to current practice. It allows to identify more HCV RNA‐positive patients (21 vs. 8), and thus leads to more HCV treatment and cure (6 vs. 3), at an acceptable cost, considering one‐time GDP per capita as a willingness‐to‐pay in France.

These results have been found in other studies, where hospital‐based screening outperforms primary care screening [17]. Hospital screening has its pitfalls. In Italian studies where the hospital HCV‐Ab prevalence was 4%–8.9% higher than in the general population, only 38%–75% of HCV‐Ab+ patients were screened for HCV RNA [18, 19]. This underlines the importance of reflex testing for HCV RNA on the same sample of HCV antibody in the event of antibody positivity, to avoid disruption of care [19]. Screening in emergency units also appears to be an effective screening strategy, but with linkage to care difficulties and very often the need for a dedicated screening team [20, 21, 22, 23]. These hospital‐based screening programmes are currently being implemented in Italy and Spain [24, 25].

The number of patients treated in the EU has been falling since 2020, making it difficult in some cases to achieve the WHO's 2030 targets [8, 24, 26, 27]. Screening strategies need to be reviewed and discussed. HCV elimination seems to be hard to achieve by universal screening in the general population even if the model suggests that it is the most cost‐effective approach [11, 28]. A study done in the ‘Baby Boomers’ population in the USA demonstrates that even with mass mailing and inclusion of a best‐practice advisory tool in electronic medical records, about 50% of baby boomers remained unscreened [13]. In France, a free screening campaign with or without prescription was set for HCV, HBV and HIV in the general population of Montpellier from September to December 2019 [12]. Among 10,143 participants, 90 (0.89%) had positive HCV‐Ab and 9 (0.09%) participants had positive HCV RNA and were treated and cured. Seventy per cent of tested participants were between 20 and 49 years old, but 83% of HCV seropositive patients were over 40 years old. In the United Kingdom, a new screening programme was created based on primary care on a birth cohort model, inviting all patients aged 40–64 to undergo an HCV‐Ab test using an oral swab kit posted to their homes [14]. Among 93,396 participants, 16.7% of patients consented and 12.4% returned a kit with 31 participants (yield 0.03%) testing positive for HCV‐Ab. Forty‐five per cent of those positive had a risk factor for HCV on their primary care record. Two (yield 0.002%) were confirmed HCV RNA positive and referred to treatment and both had HCV risk factors. Therefore, birth cohort screening should not be rolled out in primary care in countries with low HCV prevalence. Nevertheless, there are some success stories in countries with high HCV prevalence, such as the screening and treatment programme in Egypt, in which up to 8000 screening teams were able to screen 79% of the 62.5 million target participants in 1 year [29]. 1,501,307 patients were found HCV‐Ab positive and 76.5% had viremia. Ninety‐two per cent were treated and 98.8% achieved SVR. The two main reasons for this success story are solid political support and an abundant media campaign, reaching the general population through newspapers, radio, television and millions of text messages. This strategy in this context is cost effective [30]. However, in countries with low HCV prevalence, multiple micro‐elimination leading ultimately to macro‐elimination appears to be the most effective and realistic alternative solution.

Beside hospital‐based screening, other micro‐elimination programmes, in situations where HCV prevalence is high, such as psychiatric units, addiction centres, prisons or mobile units among a population of drug users, provide good cost‐effective results [31, 32, 33, 34, 35, 36, 37]. In addition, a recent study shows that in patients with mental disorders, HCV cure significantly lowered the frequency and duration of hospitalisation during the year following treatment, including in psychotic disorders subgroups, resulting in additional personal and societal benefits [38]. Some studies also suggested that micro‐elimination of HCV through test‐and‐treat strategies may be feasible and cost effective in men who have sex with men with HIV or people who inject drugs in France [39, 40, 41].

Another major finding of our study is that healthcare professionals are the main obstacle to screening. In our study, only 19.4% of the target population was screened. Considering patients screened outside the study, during the same period in the hospital, 26.4% of the target population was screened. These obstacles had already been observed in studies of emergency department HCV screening [42]. Furthermore, the survey we carried out prior to the study showed that among non‐liver specialists practicing in a tertiary hospital, 25% never screen for HCV and that healthcare professionals' knowledge of the risk factors for transmission of HCV could be improved [43]. A Spanish study shows that a 1‐h training session can temporarily improve screening [44].

These results show that if hospital‐based screening is to be effective, it must be automatic for all patients, with systematic reflex testing for viremia, and methodic organisation of the link between the viremic patient and the local hepatology teams, as suggested by an Italian team [18], and successfully implemented in Madrid [17].

The benefit of screening in the hospitalised population must be tempered by the fact that the care of these patients is often difficult. Of the 29 viremic patients, 38% were unaware of their diagnosis. Nine patients (31%) were successfully treated, but two died within a year due to comorbidities. Moreover, 19/29 (65%) of the patients diagnosed died within a year of inclusion due to comorbidities, reflecting the fact that this hospitalised population is often older and more prone to severe comorbidities. However, it is this older population that deserves to be screened and that is insufficiently screened in France, as shown by the study by Brouard et al. [8].

Our study has some limitations. First, the great variability of screening between departments, with the prevalence of anti‐HCV antibodies higher than in the general population (except in endocrinology), reflects the lack of willingness of some physicians to screen despite the multiple information campaigns. Second, certain departments, such as the maternity wards and emergency wards, were not included in the study. For the maternity department, HCV screening has been implemented despite the absence of recommendations to date (as is HBV screening), whereas in the emergency department, it seemed impossible because of the additional workload involved. Finally, the economic analysis is limited to the short term but provides additional insight into the value of universal screening. Performing an accurate cost‐effectiveness analysis would require a different and specific design, as well as modelling of clinical outcomes. However, since universal screening involves immediate costs to avoid potential future costs, and since this strategy appears to be cost effective in the short term, it is predictable that it will also be cost effective in the long term.

In conclusion, HCV screening in the population of patients admitted to a tertiary general hospital is a good opportunity because the prevalence of chronic HCV infection is higher, even though these patients are older and often have comorbidities at the forefront. This screening appears to be cost effective. However, the major obstacle to this screening is the caregivers. To stay on track with HCV elimination goals, large‐scale and repeated awareness campaigns among healthcare professionals seem mandatory. Furthermore, to overcome this problem we need to automate this screening with systematic reflex testing and to create direct hepatological therapeutic management links independent of non‐hepatologist caregivers.

## Author Contributions

All authors were involved in the manuscript and were accountable for all aspects of the works and approved the final manuscript. Protocol conceptualisation: S.N.S.A., M.B., L.L., G.V. and G.P.; methodology: S.D.‐B., G.P., M.B. and S.N.S.A.; funding acquisition: M.B.; data collection: S.N.S.A., S.B., M.M., S.T., G.V., V.O., C.D., F.S., O.P., P.C., X.A. and M.B.; data analysis: G.P., S.D.‐B. and M.B.; writing: M.B., S.D.‐B., G.P., C.C. and S.N.S.A.

## Conflicts of Interest

S.N.S.A., S.B., F.S. and O.P. received grant/research support from Gilead and AbbVie. S.B., G.P., S.D.‐B., M.M., L.L., G.V., S.T. and C.C.: None. P.C. provides consultancy to Gilead, AbbVie and Cooks. X.A. is a board member and provides consultancy to Bayer, Ipsen, Eisai, Servier, Gilead and Roche. M.B. received grant/research support from Gilead and AbbVie, received Honoria and consulting fees from AbbVie, Gilead, Janssen, GSK and Roche, and participated in a company‐sponsored speakers bureau from AbbVie, Gilead and Intercept.

## Data Availability

The data that support the findings of this study are available on request from the corresponding author. The data are not publicly available due to privacy or ethical restrictions.
